# Hepatitis E and Lymphocytic Leukemia in Man, Italy

**DOI:** 10.3201/eid1912.130521

**Published:** 2013-12

**Authors:** Maria T. Giordani, Paolo Fabris, Enrico Brunetti, Sam Goblirsch, Luisa Romanò

**Affiliations:** San Bortolo Hospital, Vicenza, Italy (M.T. Giordani, P. Fabris); University of Pavia, Pavia, Italy (E. Brunetti);; Winona Health, Winona, Minnesota, USA (S. Goblirsch);; University of Milan, Milan, Italy (L. Romanò)

**Keywords:** hepatitis E, hepatitis, hepatitis E virus, HEV, viruses, lymphocytic leukemia, immunocompromised patient, ribavirin, antiviral agents, human, chronic disease, Italy

**To the Editor:** Hepatitis E is an enterically transmitted infection with worldwide distribution and high prevalence in developing countries. This disease can occur as large water-borne epidemics associated with hepatitis E virus (HEV) genotypes 1 and 2. Hepatitis E is less common in industrialized countries, including Italy (*1*), where sporadic autochthonous cases associated with genotypes 3 and 4 have been reported. Virus strains of these genotypes are widespread in different mammalian species, including wild boar (*2*).

We report a case of hepatitis E in a 60-year-old man born and living in Vicenza, Italy, who was admitted to the Emergency Department of Vicenza Hospital on May 9, 2012 with symptoms of acute icteric hepatitis. He had been given a diagnosis of chronic lymphocytic leukemia and hemolytic anemia in 2003 and underwent 8 treatment cycles of cyclophosphamide and steroids, which were completed 20 days before he came to the Emergency Department.

His liver function test results at admission were the following: alanine aminotransferase 1,804 IU/L, total bilirubin 24.1 mg/dL, and alkaline phosphatase 137 IU/L. Test results for other causes of viral hepatitis were negative (Figure). A liver biopsy performed on June 1 showed severe acute lobular hepatitis with necrosis and cholestasis. Serum obtained at admission was positive for IgM and IgG against HEV (Dia.Pro Test, Milan, Italy). 

**Figure:**
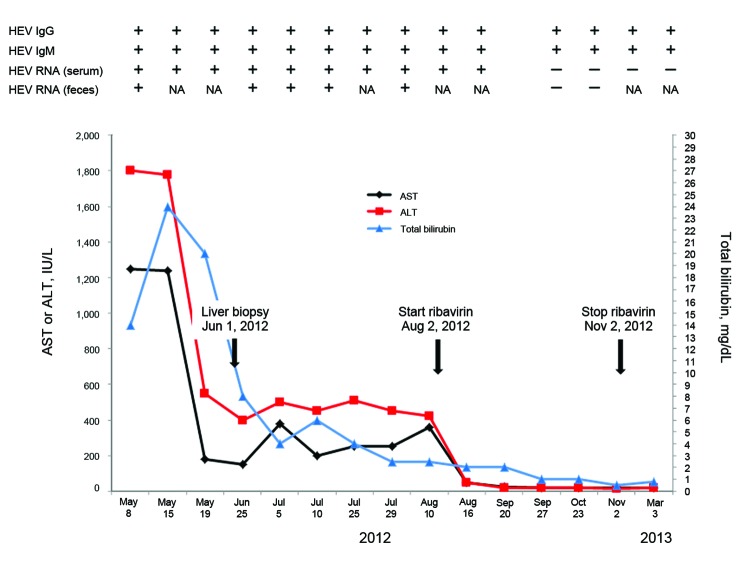
Clinical and laboratory data for a 60-year-old man with hepatitis E and lymphocytic leukemia, Italy, 2012. Start of ribavirin treatment and virologic response are indicated. A differential diagnosis was obtained by using abdominal ultrasound, which showed an enlarged hypoechogenic liver and thickening of the gallbladder wall (5 mm) with gallstones in the lumen, but a regular biliary tree. An enlarged spleen (bipolar diameter 15.07 cm) and lymph nodes were attributed to chronic lymphocytic leukemia. Test results for the following markers were negative: viral hepatitis A, B, and C; hepatitis B virus DNA (nested PCR; Roche, Switzerland); hepatitis C virus (nested PCR; Roche); cytomegalovirus virus DNA; Epstein-Barr virus; Q fever; agglutination for *Leptospira* spp. (Galton's test); enteric fever; *Borrelia* spp.; *Bartonella* spp.; autoantibodies (anti-nuclear, anti–liver kidney microsomal, anti-mitochondrial); blood and feces cultures; and ova and parasites in feces. HEV, hepatitis E virus; NA, not available; AST, aspartate aminotransferase; ALT, alanine aminotransferase. Reference ranges were 8–48 IU/L for AST, 7–55 IU/L for ALT, and 0.1–1.0 mg/dL for total bilirubin.

HEV RNA was detected by reverse transcription PCR and open reading frame 2 (*3*) was detected in serum and feces samples on May 9. Phylogenetic analysis of sequences identified HEV genotype 3 subtype h in serum and fecal samples (GenBank accession nos. KC782933 and KC782934).

Three months after admission, the patient had viremia, and results of liver function tests were abnormal. Recent data suggest that immunosuppressed persons who are viremic 3 months after HEV infection do not spontaneously clear HEV (*4*). Therefore, the patient was given antiviral therapy to achieve viral clearance. Ribavirin, 1,000 mg/day in 2 doses (400 and 600 mg), was administered during August 2–November 2, 2012. This drug was well tolerated, although the patient experienced mild anemia (hemoglobin level 10.5 mg/dL), which did not require any treatment.

Liver function test results returned to reference levels on day 14 of treatment. HEV RNA was detected in blood and feces on day 18 of treatment (August 20). Viral clearance (HEV absent from feces and serum) was achieved on day 54 of treatment (September 27) and was sustained over a 6-month period after the end of therapy.

The source of the HEV infection was uncertain. The patient had never traveled outside Italy. However, he had butchered a wild boar that he had hunted in Barberino del Mugello (Tuscany) in March 2012. The patient’s wife, who also butchered the animal, was positive for IgG against HEV but negative for IgM against HEV and for HEV RNA in February 2013. No boar meat was available for HEV testing, which indicated that this route of transmission was likely, but not confirmed.

Autochthonous hepatitis E in industrialized countries is usually an acute, self-limiting disease, but chronic disease can occur in immunocompromised hosts (*5*). These hosts include transplant recipients, persons infected with HIV, and patients with hematologic malignancies. Chronic infection with HEV has only been documented with genotype 3 strains and has been observed in many countries in Europe, However, to our knowledge, no cases of chronic infection with HEV have been reported in Italy. Our results indicate that chronic infection with HEV genotype 3 occurs in Italy.

Acute and chronic hepatitis E have been reported in patients with hematologic malignancies. An autochthonous case of acute hepatitis E was recently described in Germany in a patient with chronic lymphocytic leukemia that had been treated with chemotherapy, a bone marrow transplantation, and hemodialysis (*6*). He did not receive any specific treatment for hepatitis E and died of acute liver failure 39 days after diagnosis. Reactivation of hepatitis E in a patient with acute lymphoblastic leukemia was reported after allogeneic stem cell transplantation (*7*). HEV can also be transmitted directly from an infected transplanted organ.

Ribavirin monotherapy is an effective treatment for most patients with chronic HEV infection (*8*). It has also been used successfully to treat acute severe infection by genotype 1 of HEV in developing countries and by genotype 3 in industrialized countries (*9*), and is used to treat hepatitis C, although its mechanism of action against HCV and HEV is uncertain. Data are limited on the use of ribavirin in patients with chronic hepatitis E and hematologic malignancies (*10*). The outcome for our patient suggests that ribavirin might be useful for treating hepatitis E in such patients.

In conclusion, all patients with hepatitis of unknown origin should be tested for HEV, in particular, immunocompromised patients, because they are at risk of acquiring chronic hepatitis and having an adverse outcome. Ribavirin appears to be efficacious in treating hepatitis E and should be considered for any immunocompromised person who has viremia 3 months after acute infection.
